# Characterization of the Catalytic Structure of Plant Phytase, Protein Tyrosine Phosphatase-Like Phytase, and Histidine Acid Phytases and Their Biotechnological Applications

**DOI:** 10.1155/2018/8240698

**Published:** 2018-03-11

**Authors:** Alex Sander Rodrigues Cangussu, Deborah Aires Almeida, Raimundo Wagner de Souza Aguiar, Sidnei Emilio Bordignon-Junior, Kelvinson Fernandes Viana, Luiz Carlos Bertucci Barbosa, Edson Wagner da Silva Cangussu, Igor Viana Brandi, Augustus Caeser Franke Portella, Gil Rodrigues dos Santos, Eliane Macedo Sobrinho, William James Nogueira Lima

**Affiliations:** ^1^Engenharia de Bioprocessos e Biotecnologia e Programa de Pos-Graduação em Biotecnologia, Universidade Federal do Tocantins, Gurupi, TO, Brazil; ^2^Engenharia e Ciências de Alimentos, Universidade Estadual Paulista, São José do Rio Preto, SP, Brazil; ^3^Laboratório de Biologia Molecular e Bioquímica-ICVN, Universidade Federal da Integração Latino-Americana, Foz do Iguaçu, PR, Brazil; ^4^Engenharia de Bioprocessos e Biotecnologia, Instituto de Recursos Naturais, Universidade Federal de Itajubá, Itajubá, MG, Brazil; ^5^Faculdade de Medicina, Universidade Estadual de Montes Claros, Montes Claros, MG, Brazil; ^6^Instituto de Ciências Agrárias, Universidade Federal de Minas Gerais, Montes Claros, MG, Brazil; ^7^Instituto Federal do Norte Minas Gerais, Araçuaí, MG, Brazil

## Abstract

Phytase plays a prominent role in monogastric animal nutrition due to its ability to improve phytic acid digestion in the gastrointestinal tract, releasing phosphorus and other micronutrients that are important for animal development. Moreover, phytase decreases the amounts of phytic acid and phosphate excreted in feces. Bioinformatics approaches can contribute to the understanding of the catalytic structure of phytase. Analysis of the catalytic structure can reveal enzymatic stability and the polarization and hydrophobicity of amino acids. One important aspect of this type of analysis is the estimation of the number of *β*-sheets and *α*-helices in the enzymatic structure. Fermentative processes or genetic engineering methods are employed for phytase production in transgenic plants or microorganisms. To this end, phytase genes are inserted in transgenic crops to improve the bioavailability of phosphorus. This promising technology aims to improve agricultural efficiency and productivity. Thus, the aim of this review is to present the characterization of the catalytic structure of plant and microbial phytases, phytase genes used in transgenic plants and microorganisms, and their biotechnological applications in animal nutrition, which do not impact negatively on environmental degradation.

## 1. Introduction

Biocatalysts are important molecules that improve fermentable substrate availability and nutrient absorption, increasing the efficiency of food bioconversion. Phytases, proteases, xylanases, and amylases are typical examples of enzymatic groups that have wide applicability and contribute to increased productivity and profitability [1–3]. In monogastric animals, such as pigs, poultry, and humans, there is little or no phytase activity [4, 5]. However, these animals are fed with seeds and grains rich in phytate, which is not completely absorbed. Thus, phytate is excreted in the feces, increasing the environmental pollution by phosphorus [6, 7].

Sustainable agriculture can be achieved by adding phytase to animal feeds. Phytase improves the quality and bioavailability of some important nutrients, reducing the environmental impact of farming at the animal feed stage [8–10]. Thus, several strategies can be explored with the aim of achieving bioprocesses that increase yields and phytase stability by using microorganisms and plants. Among the microorganisms capable of producing phytase are bacteria, such as* Lactobacillus*,* Escherichia*,* Bacillus*,* Xanthomonas*,* Pseudomonas*, and* Klebsiella* spp.; fungi, such as* Penicillium*,* Aspergillus*, and* Rhizopus* spp.; and yeasts, such as* Saccharomyces cerevisiae*,* Schwanniomyces castellii*,* Schizophyllum commune*,* Wickerhamomyces anomalus*, and* Hansenula* spp. [11–13].

Plant growth and productivity can be hindered by low availability of phosphorus (P) in the soil [14]. Therefore, plants have developed mechanisms to improve soil phosphorus availability, with emphasis on the participation of purple acid phosphatases (PAPs). PAPs are acidic metallohydrolases that are involved in plant growth and pathogen defense. PAP-like isoforms have been identified in* Ipomoea batatas*,* Solanum lycopersicum*, and* Hordeum vulgare*. In barley, PAPs are grouped into isogenes HvPAPhy_a, HvPAPhy_b1, and HvPAPhy_b2 [15–17]. Another consideration is the technical compatibility of microbial phytase genes and plants, which enables the generation of transgenic crops. Understanding of the catalytic structure and amino acid sequences of microbial phytases and plants, via analysis of the principal genes, allows for technological advances using phytase without increasing environmental degradation.

Thus, the aim of this review is to present the characterization of the catalytic structure of plants and microbial phytases and their biotechnological applications, while proposing technical alternatives for sustainable agricultural productivity.

## 2. Phytase and Phosphorus Bioavailability

**Figure 1:**
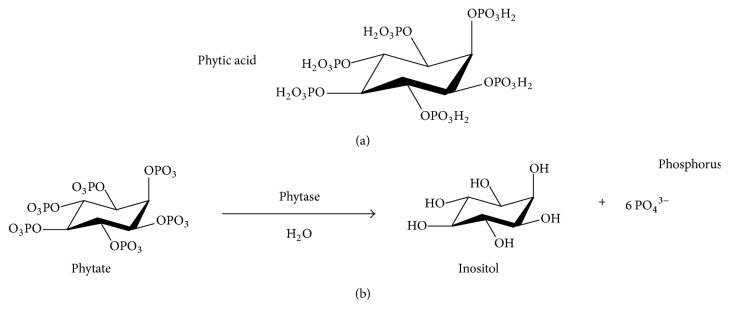
Molecular structures. The phytic acid (a) and mechanism of enzyme action (b) developed by ChemBioDraw Ultra version 12 software.

Phytate (C_6_H_18_O_24_P_6_), also known as phytic acid (myo-inositol 1,2,3,4,5,6-hexakisphosphate; PA; IP_6_), has a molecular weight of 660 g mol^−1^ (Figure 1) and is considered the main source of stored phosphorus in seeds, grains, and vegetables. Phytate is known as a food inhibitor due to its ability to chelate and thus decreases the bioavailability of some important micro- and macronutrients, such as Zn, Mg, Mn, Fe, and Ca, in monogastric animals [18, 19].

Phytate is a phosphorus storage molecule and a constituent of cereals and grains. It has relatively low bioavailability due to its strong adsorption onto soil and unavailability for degradation by soil microorganisms [20, 21]. Plants have developed a variety of mechanisms to overcome this problem, such as upregulation of high-affinity phosphate transporters, improvement of internal phosphatase activity, and secretion of organic acids and phosphatases [22–25]. The latter is an important aspect as it allows mineralized organic phosphorus to be released as inorganic phosphorus into the soil [26]. Phytases are special phosphatase enzymes that catalyze the hydrolysis of phytate into lower inositol phosphates and inorganic phosphorus (Figure 1).

Human beings are limited in their ability to hydrolyze phytate; consequently, phytic acid reduces the amount of minerals required for tissue function and cellular metabolism maintenance [27]. Pigs and poultry are unable to metabolize phytate because they lack the enzyme phytase in their gastrointestinal tract; therefore, it is necessary to add inorganic phosphorus to animal feeds. However, a large amount of phytate and inorganic phosphorus are excreted into the environment because they are not fully absorbed by these animals, causing environmental impacts such as eutrophication of surface waters of lakes and rivers, harmful algal blooms, nitrous oxide production (N_2_O; greenhouse gas), growth of toxin-producing microorganisms, and the death of several aquatic species [18, 28]. Thus, it is desirable to use new techniques that aim to increase the quality and bioavailability of phosphorus in animal feed.

## 3. Characterization of Phytase

### 3.1. Sources of Phytase

Plants, animals, and microorganisms can produce phytase. In plants, it is present in wheat, barley, peas, soybeans, corn, rice, and spinach. The blood of vertebrates such as fish, sea turtles, and reptiles also contains phytase, which is produced by microorganisms such as yeasts, bacteria, and filamentous fungi [18, 19]. Microbial phytase is of great interest to industry due to its high level of production and extracellular activity, for example, in* Aspergillus* spp. [18]. Phytase (myo-inositol hexakisphosphate phosphohydrolase, EC 3.1.3.26 and EC 3.1.3.8) is characterized as a homodimeric enzyme [29, 30]. It is a phosphatase that initiates the sequential release of phytate orthophosphate groups (myo-inositol 1,2,3,4,5,6-hexakisphosphate). It belongs to the class of hydrolases and thereby hydrolyzes phytate (phytic acid) to release inositol phosphates, phosphorus, inositol, and other essential nutrients required for absorption (Figure 1). Moreover, it is an important component of a variety of metabolic processes as released phosphorus favors development, formation and mineralization of animal bone, cellular metabolism, and protein synthesis [31–35].

### 3.2. Catalytic Structure of Plant Phytase

Many enzymes with phytase activity can be obtained or expressed in plants, animals, and microorganisms [18, 36, 37]. They are classified into the following four groups according to their catalytic mechanisms: histidine acid phosphatases (HAPs), PAPs, Cys or *β*-helix phosphatases, and protein tyrosine phosphatase-like enzymes (PTP) [38].

Phytases are composed of several phosphatases; however, only some have sufficient phytase activity. The PAPs are acidic metallohydrolases, and their structures are involved in plant growth and pathogen defense. PAPs have a metal center constituting iron, zinc, and manganese [17]. The metal center coordinates the binding site residues with those of other termini. These regions are characterized by seven conserved amino acid residues in five conserved motifs (in italic):* D*XG, G*D*XX*Y*, GN*H*(D/E), VXX*H*, and G*H*X*H*. These are involved in the dimetal nuclear center coordination [17, 39–42].

**Figure 2:**
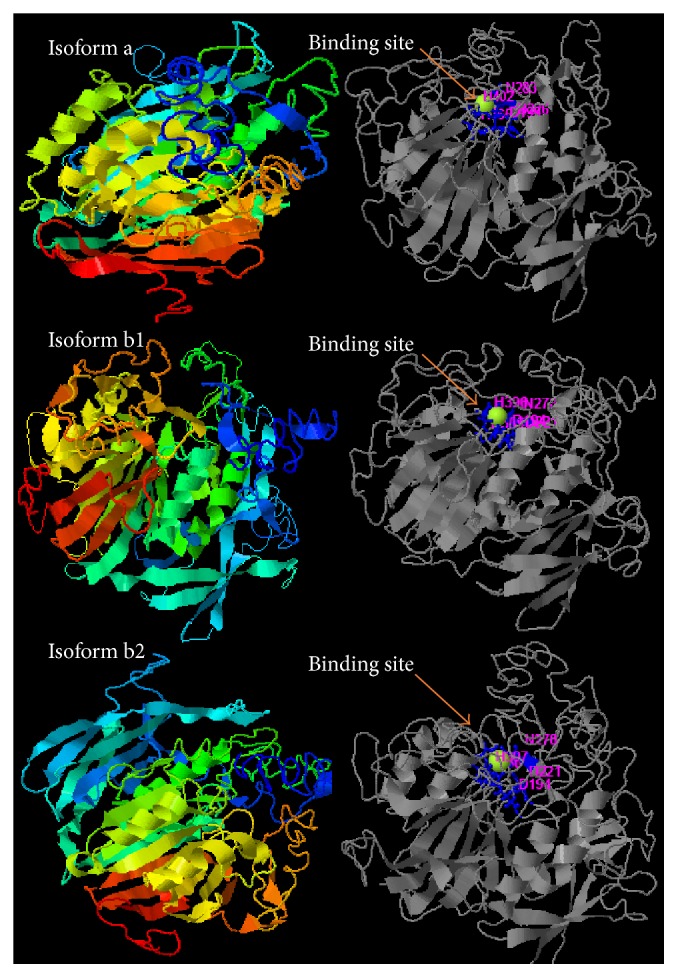
Structural model of purple acid phosphatase from barley* (Hordeum vulgare)* proposed by Dionisio et al. [4]. PAPhy genes are grouped in isogenes HvPAPhy_a, HvPAPhy_b1, and HvPAPhy_b2. Isogenes HvPAPhy possess significant phytase activity in the mature grains and proteins already were produced in* P. pastoris*. Structural model used i-TASSER server for protein structure and function prediction (https://zhanglab.ccmb.med.umich.edu/I-TASSER/) [105]. FASTA sequences were obtained from https://www.ncbi.nlm.nih.gov/protein. Isoform a is constituted by 544 amino acids, 60,29 kDa, and Hphob of 49,5%. The ligand-binding site residues from isoform a are represented by amino acid sequence 199, 226, 283, 365, and 402. Isoform b1 is constituted by 536 amino acids and 59,51 kDa with the ligand-binding site residues being represented by amino acid sequence 194, 221, 278, 359, and 396. Isoform b2 is constituted by 537 amino acids and 59,34 kDa. Ligand-binding site residues are constituted by amino acid sequence 194, 221, 278, 360, and 397.

Other PAP-like isoforms have been identified in* I. batatas* and* S. lycopersicum *[15–17]. PAPhy genes in* H. vulgare* are grouped into isogenes: HvPAPhy_a, HvPAPhy_b1, and HvPAPhy_b2 [4]. The isogene HvPAPhy possesses remarkable phytase activities in mature grains and proteins produced by* Pichia pastoris*. Isoform a (HvPAPhy_a) has a molecular mass of 60,29 kDa and contains 544 amino acids, with the ligand-binding sites at amino acid sequence 199, 226, 283, 365, and 402. Isoform_b1 (HvPAPhy_b1) has a molecular mass of 59,51 kDa and contains 536 amino acids, with the ligand-binding sites at amino acid sequence 194, 221, 278, 359, and 396. Isoform b2 (HvPAPhy_b2) has a molecular mass of 59,34 kDa and contains 537 amino acids, with the ligand-binding sites at amino acid sequence 194, 221, 278, 360, and 397 (Figure 2). The genes HvPAPhy_a, HvPAPhy_b1, HvPAPhy_b2, PHY_US417, PHYA, AVPIDOX, MtPHY1, MtPT1, and SK-57 are the major genes evaluated in transgenic plants and microorganisms presenting phytase activity (Table 1).

### 3.3. Catalytic Structure of Microbial Phytase

**Figure 3:**
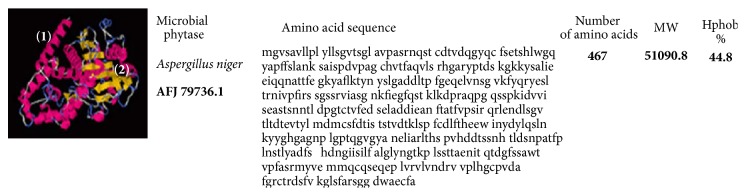
Structural model of* Aspergillus niger *phytase proposed by Mishra et al. [50] available at https://www.ncbi.nlm.nih.gov/protein and i-TASSER server used for protein structure and function prediction (https://zhanglab.ccmb.med.umich.edu/I-TASSER/) [105]. The domains are identified in the secondary structure elements being alpha-helices (1) and the secondary structure (2).

Bioinformatics approaches have contributed to increased knowledge about phytase [43]. One approach is comparative sequence analysis of phytase families, which allows for phylogenetic inferences and the prediction of functional sites [4, 34, 44]. Another is molecular modeling, which allows for inferences about enzymatic structure when no three-dimensional structure has been determined [45–47]. Based on the above-mentioned analyses, phytase B from* Aspergillus niger* (histidine acid phosphatases (HAP)) plays an important role in phytate hydrolysis. Its structure includes 460 amino acid residues and is composed of a large *α*/*β*-domain with a six-stranded *β*-sheet surrounded by several *α*-helices and a small *α*-domain. It contains five disulfide bonds at positions 52–368, 109–453, 197–422, 206–279, and 394–402, most of which are located in loops next to the surface. These bonds are due to the conformational stability of native phytases A and B and the maintenance of their catalytic activities. Moreover, researchers found that disulfide bonds have an important role in maintaining active site integrity [48]. Oakley [49] presented a structural phytase model of phytase A from* A. niger*, consisting of an *α*/*β*-domain, an *α*-domain, and an N-terminal extension. N-Acetylglucosamine residues are bound to four sites of the phytase structure (N82, N184, N316, and N353) within the active site, which is formed by an *α*-helix cavity. A structural model of* A. niger* phytase (HAP) proposed by Mishra et al. [50] is represented in Figure 3.

However, PhyA from* Xanthomonas oryzae *pv.* oryzae* (beta propeller phytase (BPHY)) plays a role in the degradation of phytic acid. It encodes a 373-amino acid protein including a 28-amino acid-predicted signal peptide. Its active site is located on the top of the *β*-propeller having a high conservation of amino acid residues involved in the metal ions binding to a phytase identified from* Bacillus amyloliquefaciens* (BaPhy), which has high- and low-affinity calcium sites responsible for enzymatic activity. PhyA from* X. oryzae *is similar to a phytase identified from* Bacillus amyloliquefaciens* (BaPhy), which has high- and low-affinity calcium sites responsible for enzymatic activity. However, differences in enzymatic activity between phytases can be attributed to differences in phosphate binding affinity [12].

**Figure 4:**
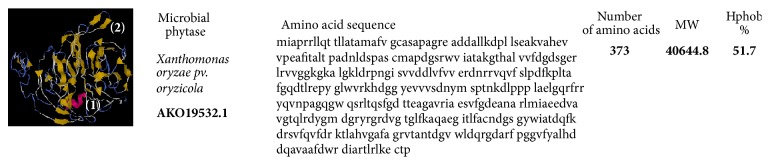
Structural model of* Xanthomonas oryzae* phytase proposed by Wilkins et al. [53] available at https://www.ncbi.nlm.nih.gov/protein and i-TASSER server used for protein structure and function prediction (https://zhanglab.ccmb.med.umich.edu/I-TASSER/) [105]. The domains are identified in the secondary structure elements being alpha-helices (1) and the secondary structure (2).

**Figure 5:**
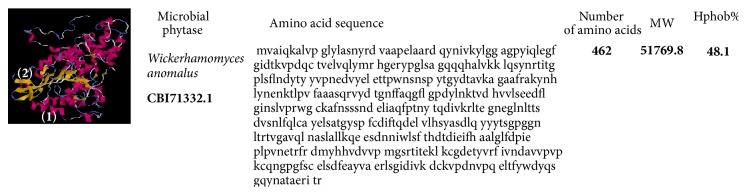
Structural model of* Wickerhamomyces anomalus* phytase proposed by Kaur et al. [54] available at https://www.ncbi.nlm.nih.gov/protein and i-TASSER server used for protein structure and function prediction (https://zhanglab.ccmb.med.umich.edu/I-TASSER/) [105]. The domains are identified in the secondary structure elements being alpha-helices (1) and the secondary structure (2).

Other types of phytases (HAP) PhyK and AppA are found in plants and microorganisms. PhyK belongs to a group of phytases synthesized by plant-associated bacteria, such as* Xanthomonas campestris*,* Pseudomonas syringae*, and* Erwinia carotovora*. This structural model was proposed by Böhm et al. [51] to explain the binding interaction between an enzyme and a substrate. The structural model observed and proposed by Shivange et al. [52] described the phytase of a mutant* Yersinia mollaretii*. Shivange et al. [52] described five amino acid substitutions, D52N, T77K, K139E, G187S, and V298M, next to the active site loop (S42-T47) and catalytically important residues involved in substrate binding (R37, R41, E241, and D327). Structural analysis of phytase from different environmental sources is important for understanding its specific role in the microenvironment [51]. Figures 4 and 5 represent structural models of* X. oryzae* phytase proposed by Wilkins et al. [53] and* W. anomalus* phytase proposed by Kaur et al. [54].

*Wickerhamomyces anomalus* has already been described as* Pichia anomala* and* Hansenula anomala*. This species exhibits antimicrobial activities and flavoring features that are responsible for its frequent association with food, beverages, and feed products [13]. Joshi and Satyanarayana [55] described phytase from* P. anomala* as a HAP that is thermostable and used as a feed additive. The PPHY gene of* P. anomala* synthesizes a cell-bound phytase with an ORF of 1389 bp encoding a 462-amino acid protein [53]. Kaur et al. [54] described that the amino acid sequence analysis from* P. anomala* has varying similarity to those of phosphatases from* Pichia stipitis* (62%),* Candida dubliniensis* (51%),* Candida albicans* (51%), and* Arxula adeninivorans* (35%) and phytases from* Debaryomyces castellii* (50%) and* Pichia fabianii* (39%). However, amino acid sequence analysis from* W. anomalus* [54] showed only 5% similarity to phytase from* A. niger* [49].

**Figure 6:**
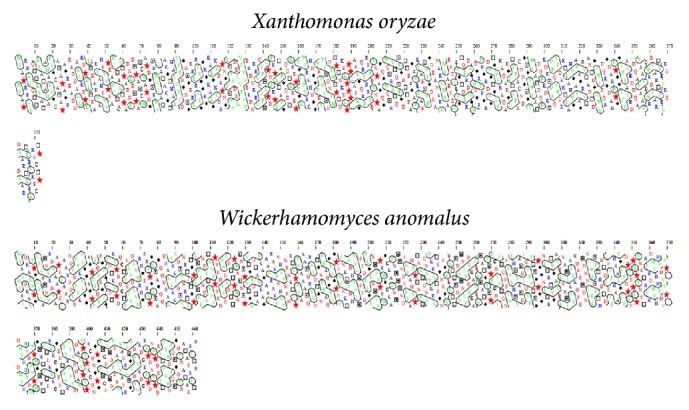
Secondary structure assignment of microbial phytase by Hydrophobicity Clusters Analysis (HCA) proposed by Bertrand et al. [57]. FASTA data proposed by Kaur et al. [54] are available at https://www.ncbi.nlm.nih.gov/protein. Hydrophobic amino acids are not distributed randomly but form clusters. The clusters are correspondent for the real positions in the regular secondary structures. Vertical clusters are often associated with beta strands and horizontal clusters correspond to alpha-helices.* Xanthomonas oryzae*, Hphob = 51,7%, and* Wickerhamomyces anomalus*, Hphob = 48,1%.

**Figure 7:**
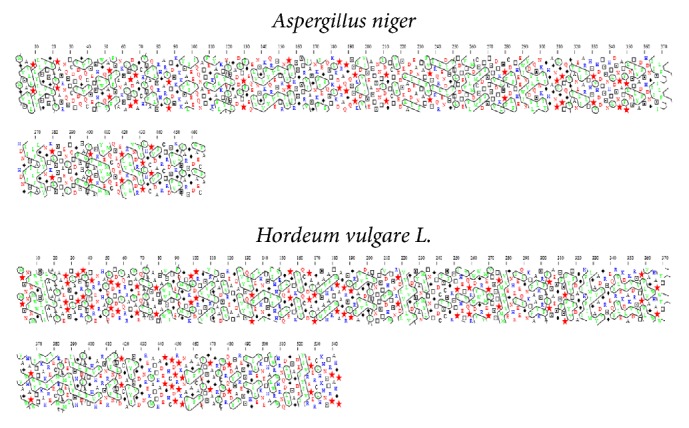
Secondary structure assignment of phytase from* Aspergillus niger* and* Hordeum vulgare *L. by Hydrophobicity Clusters Analysis (HCA) proposed by Bertrand et al. [57]. FASTA data proposed by Mishra et al. [50] and Dionisio et al. [4] are available at https://www.ncbi.nlm.nih.gov/protein. Hydrophobic amino acids are not distributed randomly but form clusters. The clusters are correspondent for the real positions in the regular secondary structures. Vertical clusters are often associated with beta strands and horizontal clusters correspond to alpha-helices.* Aspergillus niger*, Hphob = 44,8%, and* Hordeum vulgare* L. isoform *a*, Hphob = 49,5%.

Structural models of* A. niger*,* W. anomalus,* and* X. oryzae* presented here revealed varying percentages of hydrophobicity between microbial groups. The hydrophobicity of phytase from* A. niger* (44.8%) and that of phytase from* W. anomalus* (48.1%) are similar; however, that of* X. oryzae* is high and different (51.7%) (Figures 3–5). Similarly, the hydrophobicity of HvPAPhy_a is 49.5% (Figure 2). Phytases from* A. niger*,* W. anomalus*, and* H. vulgare* have low hydrophobicity compared with those from* X. oryzae*. The degree of hydrophobicity is an important aspect and reflects the proportions of *β*-sheets and *α*-helices in the protein structure. Additionally, the relative amounts of hydrophobic and hydrophilic amino acids partially explain the enzymatic stability. HAP phytase from* Sporotrichum thermophile* showed that surface charge distribution and a high density of hydrophilic amino acids on the surface contribute to the thermal stability of phytase [56]. Moreover, using bioinformatics analysis, Bertrand et al. [57] reported that hydrophobic amino acids are not distributed randomly but form clusters. Thus, it has been suggested that the position of a cluster corresponds to real positions in the secondary structures [57]. Vertical clusters are often associated with *β*-sheets, whereas horizontal ones often correspond to *α*-helices [57]. The hydrophobicity of phytases from plants and microorganisms analyzed was represented in this study by Hydrophobicity Clusters Analysis according to Bertrand et al. [57], with the aim of contributing to the analysis of phytase thermostability (Figures 6 and 7).

## 4. Bioprocess and Strategy of Production

### 4.1. Transgenic Plants and Phytase

Plant growth and productivity can be hindered by low availability of phosphorus in the soil [14]. Therefore, plants have developed mechanisms that include upregulation of high-affinity phosphate transporters and improvement of internal phosphatase activity [22–24]. Transgenic plants have been used with the aim of increasing the availability of phosphorus in the soil. Genes such as the *β*-propeller phytases from* Bacillus subtilis* have been inserted in transgenic plants of* Arabidopsis* and tobacco [14, 58].

A recent study revealed overexpression of an extracellular form of the phytase PHY_US417 of* B. subtilis* secreted by transgenic Arabidopsis lines (ePHY) and showed increased phosphate acquisition [14]. Furthermore, phytase genes PhyA and AppA from* A. niger* and* Escherichia coli* have been inserted into* Brassica napus* and increased phosphorus bioavailability in soil and seed phytate [59]. Other reports described a reduction of 50% and 48% in phosphorus secretion by broilers and piglets, respectively, after feeding with transgenic soybean* (Glycine max)* and canola* (B. napus)* seeds with reduced phytase activities [60, 61]. This strategy has also achieved success in decreasing seed phytate levels by endogenous phytase gene expression during the early stages of seed development in transgenic soybean [62]. However, it remains necessary to advance knowledge about the molecular mechanisms regulating phytase formation during grain development and germination [4].

Transgenic wheat and microalgae have been employed for the expression of phytase activity. Transgenic microalgae, such as* Chlamydomonas reinhardtii*, have been revealed as an interesting model system for the production of a fungal phytase (*A. niger* PhyA E228K, mE228K) due to their suitability for genetic transformation and scalability. Lines with high expression levels of in vitro phytase activity were obtained [63]. Expression of the PhyA gene from* Aspergillus japonicus* in wheat transgenic plants exhibited an increase in phytase activity and iron and zinc contents and a decrease in phytic acid content in seeds. Such reports strengthen an effective method of increasing the bioavailability of iron and zinc and propose a novel way to combat nutritional deficiency [64].

Thus, transgenic plants may be an alternative that warrants further exploration due to the technical compatibility of the genes of plants and microorganisms. It is a promising technology because it promotes efficiency and agricultural productivity, aiming to improve animal nutrition without causing environmental degradation.

### 4.2. Microbial Phytase

Enzyme production has been observed in various species, especially* A. niger* [20, 65, 66]. It is possible to observe variations in the yield of enzymatic production due to the physiological peculiarity of each microorganism and varying bioprocess conditions [67].* A. niger* wild-type strain NRRL3135 produces high amounts of phytase in mineral salts medium, containing glucose and corn starch as the carbon source. However, with the addition of phosphate in the culture medium formulation, there is no evidence of phytase production.* A. niger* can produce two kinds of phytase, PhyA and PhyB, depending only on the pH variation from 2.5 to 5.5, respectively. Moreover, it is possible to use genetic engineering tools to introduce the PhyA gene driven by a specific promoter into another strain of* A. niger*, producing a high yield [68].

*Bacillus amyloliquefaciens* produces extracellular phytase at 37°C in a medium supplemented with wheat bran and casein hydrolysate by submerged fermentation. Other species also produce high amounts of phytase under specific fermentation conditions.* B. subtilis* can be cultivated at 60°C and pH between 7 and 7.5 to produce high amounts of phytase. Moreover,* B. subtilis* has been used in genetic modifications aimed at the expression of specific phytase genes. Other examples are phytases from* Aspergillus awamori* and* Aspergillus fumigatus* employed by genetic methods modified and evaluated by SDS-PAGE and Western blotting. Thus, these works reveal that the recombinant strain exhibits overexpression, high activity, and thermostability at pH between 4 and 7 at 60°C [69, 70].

*Pichia pastoris* is a methylotrophic, nonpathogenic yeast and is widely used to produce various heterologous proteins because it has the capacity to perform important and necessary posttranslational modifications of the protein of interest. Transcription of the genes inserted into the vector is driven by the AOX1 promoter mediated by the action of methanol in the medium or in the presence of methanol as a sole carbon source [71, 72]. The phytase gene of 1347 bp from* A. niger* SK-57 can be obtained by PCR and expressed in* P. pastoris* using the AOX1 promoter. Under these conditions,* P. pastoris* produced 6.1 g·l^−1^ of purified phytase with an activity of 865 U·ml^−1^ and pH optima between 2.5 and 5.5 at 60°C. Thus,* P. pastoris* can be an alternative microorganism for use as a production system for commercial phytase [73]. Another strain of methylotrophic yeast,* Hansenula polymorpha*, can be genetically modified to express phytase effectively under submerged fermentation conditions with the use of the formate dehydrogenase promoter [11].

Moreover, phytase can be obtained from the mushroom* Schizophyllum* sp. Cultivation is usually carried out in solid-state fermentation at 30°C in a culture medium consisting of wheat bran suitable for a few days at pH between 6 and 7 [74]. Gram-negative bacteria of the* Chromobacterium* sp. genus have been isolated from a variety of habitats in Brazilian ecosystems and have already presented phytase activity.* Chromobacterium* sp. usually can be cultivated at 28°C in a specific medium containing phytate. Bacteria of the genera* Escherichia, Rahnella*, and* Pseudomonas* also produce phytase [75]. Different bioprocesses for microbial phytase production are described in Table 2.

## 5. Animal Supplementation and Sustainable Agriculture

In monogastric animals, there is little or no evidence of phytase activity in the digestive tract [4, 5]. In these animals, food is not completely digested, and cellular bioavailability is low. Most of the phytate present in the feed is excreted, increasing the environmental phosphate concentration in areas where livestock grazing is intense [6, 7]. Low-phosphate bioavailability and phosphorus deficiency in animal feed based on dry grain can be supplemented by adding inorganic phosphate. Due to the high market demand for meat, this strategy of phosphorus supplementation is used increasingly in animal feed. However, the practice of adding inorganic phosphorus is unsustainable, because phosphorus is not a renewable resource in nature, and its continued harvesting would result in depletion of this environmental resource in a few decades [6, 9, 67, 76, 77]. Thus, the addition of phytase to animal feed is an interesting and sustainable alternative, due to its ability to increase phosphate bioavailability in diets based on dry grain and to decrease the environmental impacts caused by phosphorus accumulation in the environment [4, 31, 78]. Adding phytase to animal feed also reduces the operational production cost as the inclusion of inorganic phosphate is no longer required. Moreover, phytase also increases the availability of other minerals such as zinc, iron, and manganese, which are important for animal metabolism [7, 67, 79–82]. Animal feed supplementation provides beneficial responses in terms of animal performance such as increased weight of ruminants, broilers, and fishes [82–84]. Other beneficial effects can be achieved by incorporating mixed enzymatic formulations that result in an increase in the coefficients of ileal nutrient digestibility and standardized ileal digestibility by amino acid absorption and energy production [85–88].

Multienzymatic complexes can also provide efficient nutrient absorption. Previous reports described secondary effects associated with the action of multienzymatic complexes added to stimulate hormone synthesis [89, 90]. Bedford and Cowieson [91] analyzed the multifactorial effects of exogenous enzymes on the intestinal microbiota and the improvement of animal performance. Phytase has positive contributions for use in animal feed supplementation; however, it is still necessary to evaluate the specific effects of carbohydrases and proteases on multienzymatic complexes [92–96]. Thus, animal characteristics, such as species or age, are important sources of variation in these studies. However, the main cause is directly associated with characteristics of animal feed in both qualitative and quantitative aspects. The nonstarch portion is diversified and large, which favors increased digestive viscosity and hinders enzymatic action. Studies have reported that this complicates the understanding of the effects of these multienzymatic complexes on animal feeds. Thus, their use and performance can be underestimated mainly due to their variation in the concentrations of enzyme and substrate [97].

Organic acids are other additives that can be supplemented in phytase formulations. Increased animal performance is caused by one or more actions: (1) a decrease in gastric pH favoring pepsin activity in the fed state; (2) immune system improvement due to microbiological selectivity, which reduces Gram-negative bacteria in favor of* Lactobacillus* proliferation in the gastrointestinal tract; and (3) improvement of both mineral and protein digestibility from food [98]. The benefits of organic acids show high interspecific diversity and are largely dependent on the profile of the gastrointestinal tract. In pigs, the gastrointestinal pH is above 6.0, and acidification is beneficial to phytase activity, due to its acidic pH optimum [99]. Thus, enzymatic formulations containing phytase generally include the organic acids, acetic, citric, fumaric, lactic, formic, and propionic acids [98–100], and the following enzymes: xylanases [87, 92–95], cellulase [101], *β*-glucanase [102], *β*-mannanase [96, 103], and proteases [104].

Therefore, the addition of supplementary phytase to the feed of monogastric animals shows many advantages, these being associated or not with multienzymatic complexes or with organic acids, providing an adequate supply of phosphorus and other minerals without damaging the environment.

## 6. Perspectives

Phytase plays an important role by releasing phosphorus and other nutrients from phytate added to monogastric animal feed and decreases the amount of this element in the environment. Its use allows the consumption of phosphorus available in grains used in animal nutrition. The catalytic structure, production, and biotechnological applications of phytase are increasingly evaluated in research to increase the understanding of three-dimensional structures and to optimize bioprocesses with the aim of increasing production and activity. Plants and different microorganisms can produce phytase, the yields being related to the bioprocess employed. Future perspectives indicate the use of recombinant plants or microorganisms with phytase activity to improve the use of phosphorus and other minerals and to propose a new strategy to improve animal feed. Thus, understanding the catalytic structure of phytase from plants or different microorganisms is an important aspect that allows for sustainable agriculture and attention to the environment.

## Figures and Tables

**Table 1:** Major genes evaluated in transgenic plants and microorganisms with phytase activity.

Gene	Plant target	Microbial target	Reference
HvPAPhy_a	*Hordeum vulgare *L.	—	Holme et al. [106]
PHY_US417	*Arabidopsis thaliana*	—	Belgaroui et al. [14]
PHYA	*Hordeum vulgare *L.	—	Mohsin et al. [107]
AVP1DOX	*Solanum lycopersicum*	—	Yang et al. [108]
MtPHY1	*Medicago sativa *L.	—	Ma et al. [109]
MtPT1	*Medicago sativa *L.	—	Ma et al. [109]
HvPAPhya	—	*Pichia pastoris*	Dionisio et al. [4]
HvPAPhyb1	—	*Pichia pastoris*	Dionisio et al. [4]
HvPAPhyb2	—	*Pichia pastoris*	Dionisio et al. [4]
*sk-57*	—	*Pichia pastoris*	Xiong et al. [73]

**Table 2:** Microbial phytase obtained by submerged and solid-state fermentation.

Microorganisms	Process	Yield	Reference
*Aspergillus niger *mutant	SSF	154.0 U·l^−1^	Bhavsar et al. [79]
*Chromobacterium *sp.	SF	7.4–12.4 U·ml^−1^	Costa et al. [75]
*Schizophyllum *sp.	SSF	55.5 U·ml^−1^	Salmon et al. [74]
*Bacillus subtilis*	SF	8.5–9.0 U·mg^−1^	Fu et al. [69]
*Bacillus laevolacticus*	SF	2.957 U·ml^−1^	Fu et al. [69]
*Aspergillusawamori and fumigatus*	SF	12.86 and 20.75 U·ml^−1^	Martin et al. [70]

*Hansenula polymorpha*	SF	13.5 g l^−1^	Mayer et al. [11]

SSF: solid-state fermentation; SF: submerged fermentation; U ml^−1^: units per milliliter; U mg^−1^: units per milligram; g l^−1^: gram per liter.
